# Time-Dependent Degradation of Naphthoquinones and Phenolic Compounds in Walnut Husks

**DOI:** 10.3390/biology11020342

**Published:** 2022-02-21

**Authors:** Aljaz Medic, Tilen Zamljen, Metka Hudina, Robert Veberic

**Affiliations:** Department of Agronomy, Biotechnical Faculty, University of Ljubljana, Jamnikarjeva 101, SI-1000 Ljubljana, Slovenia; tilen.zamljen@bf.uni-lj.si (T.Z.); metka.hudina@bf.uni-lj.si (M.H.); robert.veberic@bf.uni-lj.si (R.V.)

**Keywords:** juglone, α-hydrojuglone, hydrojuglone glucoside, oxidation, degradation, *Juglans regia*, husk

## Abstract

**Simple Summary:**

Some studies have examined the degradation of individual phenolic compounds. However, the degradation of naphthoquinones has been poorly investigated, with contradicting reports. This study investigated how individual phenolic compounds and phenolic groups (e.g., naphthoquinones, flavanols, flavonols, hydroxycinnamic acids) are oxidized over time in walnut husk gratings, to better explain, or confirm, the process of the juglone release pathway. The study was designed to initially determine whether the proposed juglone synthesis pathway of hydrojuglone glucoside → α-hydrojuglone → juglone is indeed correct, or whether an alternative pathway is seen. The study also provides new data about the degradation of individual phenolics and phenolic groups over time when damaged tissue is exposed to the air. As phenolic compounds are considered highly beneficial to human health, increases upon processing indicate the need for further investigations into healthier food preparation processes.

**Abstract:**

The aim of the present study was to investigate how individual phenolic compounds and phenolic groups in walnut husk gratings (e.g., naphthoquinones, flavanols, flavonols, hydroxycinnamic acids) are oxidized over time, with a particular focus on the juglone synthase pathway. Walnut husk gratings were prepared and left under ‘degradation’ conditions (exposure to the air, room temperature) at increasing times. Following methanol extraction of these husk gratings, the HPLC profile of methanolic extract of husk gratings exhibited twenty-six compounds over time, then hydrojuglone glucoside, α-hydrojuglone, and juglone were detailed by HPLC-mass spectrometry. Initially (0–20 min), the content of hydrojuglone glucoside in the husk gratings decreased by 40.4%, while the content of α-hydrojuglone increased by 20.0%, and then decreased. After an initial delay (0–20 min), juglone increased by 47.9% from 20 to 40 min, and then decreased. This initially confirmed that hydrojuglone glucoside and α-hydrojuglone could be considered as precursors of juglone. Different phenolic groups showed different degradation processes, although they all reached their highest content after 40 min. This might arise from degradation of the phenols, increased free phenols, or activation of the plant defense mechanism due to damage to the tissue, similar to the effects of stress or a pathogen attack. Although it has been reported that the phenolic compounds decrease when food is processed or damaged, they showed increases, which were not indefinite, but time dependent. As phenolic compounds are considered highly beneficial to human health, increases upon processing indicate the need for further investigations into healthier food preparation processes. This is the first study on the degradation pathways of juglone, using a mass spectrometer, in which we suggest that hydrojuglone glucoside and α-hydrojuglone are indeed the precursors of juglone. However, it is possible that there are other degradation pathways of hydrojuglone glucoside, since less juglone is synthesized than expected.

## 1. Introduction

Phenolic compounds are classified as secondary metabolites. In plants, they are composed of a diverse group of molecules that have a wide range of functions and structures, although they generally have an aromatic ring that bears one or more hydroxyl substituents. These phenolic compounds are considered the most important, numerous, and ubiquitous group of compounds in the plant kingdom [1]. 

Phenolic compounds are synthesized during plant development. Among their several functions in plants, they most commonly serve as part of plant defense mechanisms and are thus synthesized in various situations in response to stress, pathogen attacks, UV radiation, and other factors [2]. They are mainly classified according to the number of phenolic rings they contain, although they have also been divided according to their water solubilities: (i) water-insoluble compounds, which include lignins, condensed tannins, and the cell wall-bound hydroxycinnamic acids; and (ii) water-soluble compounds, which include quinones, flavonoids, phenylpropanoids, and phenolic acids [3].

Quinones are classified according to their specific aromatic skeleton, and from a chemical point of view, they are classified as benzoquinones, 1,4- and 1,2-naphthoquinones, furanonaphthoquinones, and anthraquinones, which are in the form of monomeric or dimeric units [4]. Most quinones belong to the groups of naphthoquinones, benzoquinones, anthraquinones, and phenanthrenequinones [5]. Of these, the naphthoquinones are of particular interest, because they occur as natural products [6]. Among the naphthoquinones, juglone (5-hydroxy-1,4-naphthoquinone) remains of great interest, because of its high chemical reactivity and allelopathic effects [7,8].

Most flavonoids in plants are glycosides and, thus, they include different sugar units and acylated sugars at different positions of the polyphenol skeleton [9]. When flavonoids are associated with one or more sugar molecules, they are known as flavonoid glycosides, and when they are not associated with a sugar molecule, they are known as aglycones. In general, the aglycone forms are more active than the glycoside forms, although glycosylated flavonoids are more common in plants [10]. The flavonoids are the most important of the bioactive compounds in plants, and they have been divided into six subclasses according to their chemical structures: flavan-3-ols, flavanones, flavones, flavonols, isoflavones, and anthocyanins [3].

**Figure 1:**
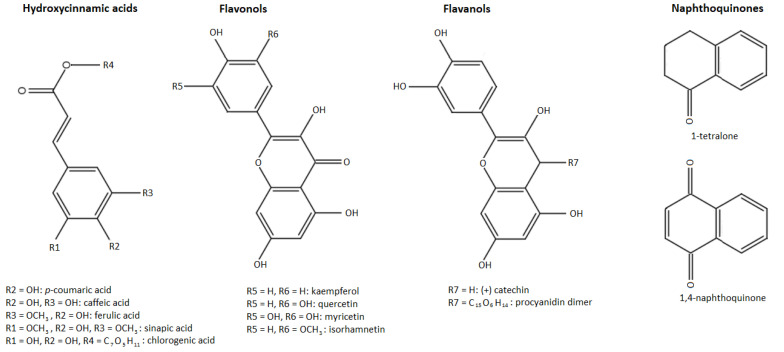
Phenolic groups identified in walnut husk gratings.

The second most important group of these phenolic compounds are the phenolic acids, which are present in plants in bound, rather than soluble, forms. The phenolic acids have been divided into two subgroups: hydroxybenzoic acids and hydroxycinnamic acids. Unlike the other phenolic compounds, these are acidic in character because they contain a carboxyl group. Hydroxycinnamic acids occur mainly as derivatives with a C6–C3 skeleton, whereas hydroxybenzoic acids have a C6–C1 skeleton and occur mostly as esters [9,11]. Visualization of different phenolic groups studied in our research can be observed in Figure 1.

Considering plants and foods in general, under stress conditions, plants respond with an increase in phenolic compounds, but once the fruit is removed from the plant, this response would not be expected to continue in the fruit itself [12]. However, for vegetables, the studies carried out have been inconclusive, or even contradictory, as the phenolic content of vegetables can increase over time, or after thermal treatments or a pathogen attack; alternatively, the phenolic content can also decrease over time [13,14,15]. Thermal treatments of various food matrices have been reported to promote the release of bound phenols and, thus, lead to an increase in the free phenolic fraction [16]. The increase in phenolic content in vegetables might be due to the degradation of phenols [17] or to the increase in free flavonols [18]. 

Some studies have examined the degradation of individual phenolic compounds [19,20,21,22]. However, the degradation of naphthoquinones has been poorly investigated, with contradicting reports [8,23,24]. As juglone is the most common and widely known naphthoquinone and one of the first allelochemicals discovered [7], it is also the focus of the present study. The biosynthesis of juglone remains to be fully elucidated, although the shikimate pathway has been suggested as the likely candidate, since several important precursors contain 1,4-naphthoquinone, *o*-succinylbenzoic acid, and 2-succinylbenzoate [25]. 

As with most plants that produce toxic secondary metabolites, juglone is stored in a nontoxic form in the tree [25]. In 1943, it was first demonstrated that juglone is not present in the root, bark, and husks of walnut, but is instead in the form of α-hydrojuglone, which is nontoxic, but is oxidized to juglone when exposed to air [26]. Less than a decade later, it was shown that juglone is stored in walnut as the 5-glucoside of 1,4,5-tri-hydroxynaphthalene [27]. This glucoside is very labile and easily hydrolyzed to glucose and α-hydrojuglone. The conversion of the glucoside to juglone was proposed to be a two-step process, with β-glucosidase (a common soil enzyme) catalyzing its hydrolysis in the first step, followed by rapid chemical oxidation in the second step [8]. However, as indicated, this conversion has been poorly studied, and the few studies carried out have reported conflicting results. 

The aim of the present study was both to investigate how individual phenolic compounds and phenolic groups (e.g., naphthoquinones, flavanols, flavonols, hydroxycinnamic acids) are oxidized over time, and to better explain, or confirm, the process of the juglone release pathway. The study was therefore designed to initially determine whether the proposed juglone synthesis pathway of hydrojuglone glucoside → α-hydrojuglone → juglone is indeed correct, or whether an alternative pathway is seen. The study also provides new data on various phenolic compounds, in terms of the degradation of individual phenolics and phenolic groups over time when damaged tissue is exposed to the air.

## 2. Materials and Methods

### 2.1. Plant Material

Samples of *Juglans regia* L. (walnut) husks were obtained on 22 September 2021, from a 25-year-old walnut tree of the French cultivar ‘Franquette’. The planting density of the trees was 10 m × 10 m at the Experimental Field for Nut Crops in Maribor (Slovenia; 46°34′01″ N; 15°37′51″ E; 275 m a.s.l.). The samples were collected from one tree, from the middle third of the branches on the eastern side, to obtain material that was as uniform as possible. The samples were taken to the laboratory for further analysis (Department of Agronomy, Biotechnical Faculty, University of Ljubljana, Ljubljana, Slovenia).

### 2.2. Sampling and Extraction of Individual Phenolic Compounds

Twenty healthy and undamaged walnut husks were used for the experiment. Each of the husks was grated using a standard kitchen grater (hole size, 2 mm), to obtain uniformly damaged husk tissue gratings to maintain the most uniform conditions possible for degradation (i.e., oxidation) of the phenolic compounds. The gratings from the husks were then spread evenly in a 5-mm-thick layer on a plastic cutting board (25 × 35 cm) and divided into 28 similarly-sized samples; these were left exposed to the air at room temperature. Four of the samples were randomly selected for each sampling time (i.e., four replicates per timepoint), as immediately after grating (0 min), and at 20, 40, 60, 180, 360, and 540 min exposure to degradation (in air, at room temperature). This provided an evaluation of the rate of degradation of each phenolic compound and of the different phenolic groups over time. At these specified times, the samples were placed in liquid nitrogen to prevent further degradation, and then ground using a mortar prior to the methanol extraction.

The phenolic compounds were extracted following the protocol previously described by Medic et al. [28]. Briefly, 0.25 g of husk gratings was extracted using absolute methanol (1:20, *w*/*v*, respectively). The samples were vortexed (Top-Mix 94500; Heidolph, Schwabach, Germany) for 30 s, and then sonicated (Sonis 4; Iskra pio, Sentjernej, Slovenia) in iced water for 60 min. Following the extraction, the samples were centrifuged (5810 R; Eppendorf, Hamburg, Germany) at 10,000× *g* for 10 min at 4 °C, filtered through 0.2-µm polyamide filters (Chromafil AO 20/25; Macherey-Nagel, Düren, Germany), and transferred to vials for further analysis.

### 2.3. Identification and Quantification of the Phenolic Compounds Using HPLC and Mass Spectrometry

The initial identification of all 26 of the phenolic compounds was carried out using UHPLC (Vanquish; Thermo Scientific, Waltham, MA, USA), coupled with tandem mass spectrometry (LTQ XL; Thermo Scientific, Waltham, MA, USA) with heated electrospray ionization. 

For the separation of the compounds through UHPLC, a C18 column (Gemini; Phenomenex, Torrance, CA, USA) was used with a flow rate of 0.6 mL/min, under similar conditions to those previously described [28]. The solvents used were: A, 0.1% formic acid with 3% acetonitrile in bi-distilled water (*v*/*v*/*v*); B, 0.1% formic acid with 3% bi-distilled water in acetonitrile (*v*/*v*/*v*). The gradient used was as follows: 0–15 min, 5–20% B; 15–20 min, 20–30% B; 20–25 min, 30–50% B; 25–30 min, 50–90% B; 30–35 min, 90–100% B; 35–45 min, 100–5% B; 45–50 min, 5% B. 

The mass spectrometer was operated in negative ion mode, with the following parameters used [28]: sheath temperature, 150 °C; sheath gas, 30 arb; auxiliary gas, 20 arb; ion spray voltage, 3.2 kV; capillary temperature, 300 °C; capillary voltage, −23.99 V; and tube lens, −57.36 V; collision gas, helium; collision energy, 35 eV; scans performed, from *m*/*z* 50 to 2000; data acquisition, Xcalibur 2.2 software (Thermo Fisher Scientific Institute, Waltham, MA, USA). The UHPLC-separated compounds were fragmented and identified according to data shown previously [29,30].

For the quantification over time of the phenolic compounds, due to the low content of hydrojuglone glucoside, α-hydrojuglone, and juglone in the extracted samples, and to provide their precise quantification, these were analyzed using the UHPLC–mass spectrometry system (Vanquish; LTQ XL). The remaining compounds were quantified using the UHPLC system with a photodiode array detector at 280 nm. Where commercial standards were available, the phenolic compounds are expressed according to their standard; otherwise, the nearest available standard was used. The total analyzed phenolic content represents the sum of all of the individually quantified phenolic compounds.

### 2.4. Chemicals

The following standards were used for these analyses: quercetin-3-glucoside and procyanidin B1 (Fluka Chemie GmbH, Buchs, Switzerland); (+)catechin (Roth, Karlsruhe, Germany); (−)epicatechin, quercetin-3-rhamnoside, quercetin-3-galactoside, neochlorogenic acid (3-caffeoylquinic acid), juglone (5-hydroxy-1,4-naphthoquinone), and gallic acid (Sigma–Aldrich Chemie GmbH, Steinheim, Germany); quercetin-3-xyloside, quercetin-3-arabinopyranoside, and quercetin-3-arabinofuranoside (Apin Chemicals, Abingdon, UK).

The chemicals for the mobile phases (acetonitrile, formic acid) were HPLC-MS grade, and the absolute methanol used for the extractions was HPLC grade (all from Fluka Chemie GmbH, Buchs, Switzerland). The water used was bi-distilled and purified through a Milli-Q water purification system (Millipore, Bedford, MA, USA).

### 2.5. Statistical Analysis

Data were analyzed using Microsoft Excel 2016 and R commander, version 2.7.1 (package Rcmdr) (Team R.D.C., 2008, Stanford, CA, USA). Data are expressed as means ± standard errors. One-way analysis of variance (ANOVA) with Tukey tests were used to detect significant differences between data. Statistical means were calculated at a 95% confidence level.

## 3. Results and Discussion

### 3.1. Identification of Individual Phenolic Compounds

The 26 compounds identified are given in Table 1, along with their MS^2^ fragments and the standards that were used for their quantification.

Juglone (5-hydroxy-1,4-naphthoquinone) was identified using a juglone standard according to its fragmentation pattern MS *m*/*z* 173 [M − H]^−^ and MS^2^ *m*/*z* 155 [M − H − H_2_O]^−^, 145 [M − H − CO]^−^, 129 [M − H − CO_2_]^−^, and 111 [M − H − CO_2_ − H_2_O]^−^. Hydrojuglone glucoside MS *m*/*z* 337 defined the loss of a hexosyl moiety (−162) and was identified according to the specific fragmentation pattern of α-hydrojuglone (1,4,5-trihydroxy-1,4-naphthoquinone) MS *m*/*z* 175 [M − H]^−^ and MS^2^ *m*/*z* 157 [M − H − H_2_O]^−^, and 147 [M − H − CO]^−^, and 131 [M − H – CO_2_]^−^ [30,31]. The fragmentation patterns of the remaining compounds can be found in the previous studies by Medic et al. [29,30,32].

### 3.2. Degradation of the Individual Phenolic Compounds over Time

**Figure 2:**
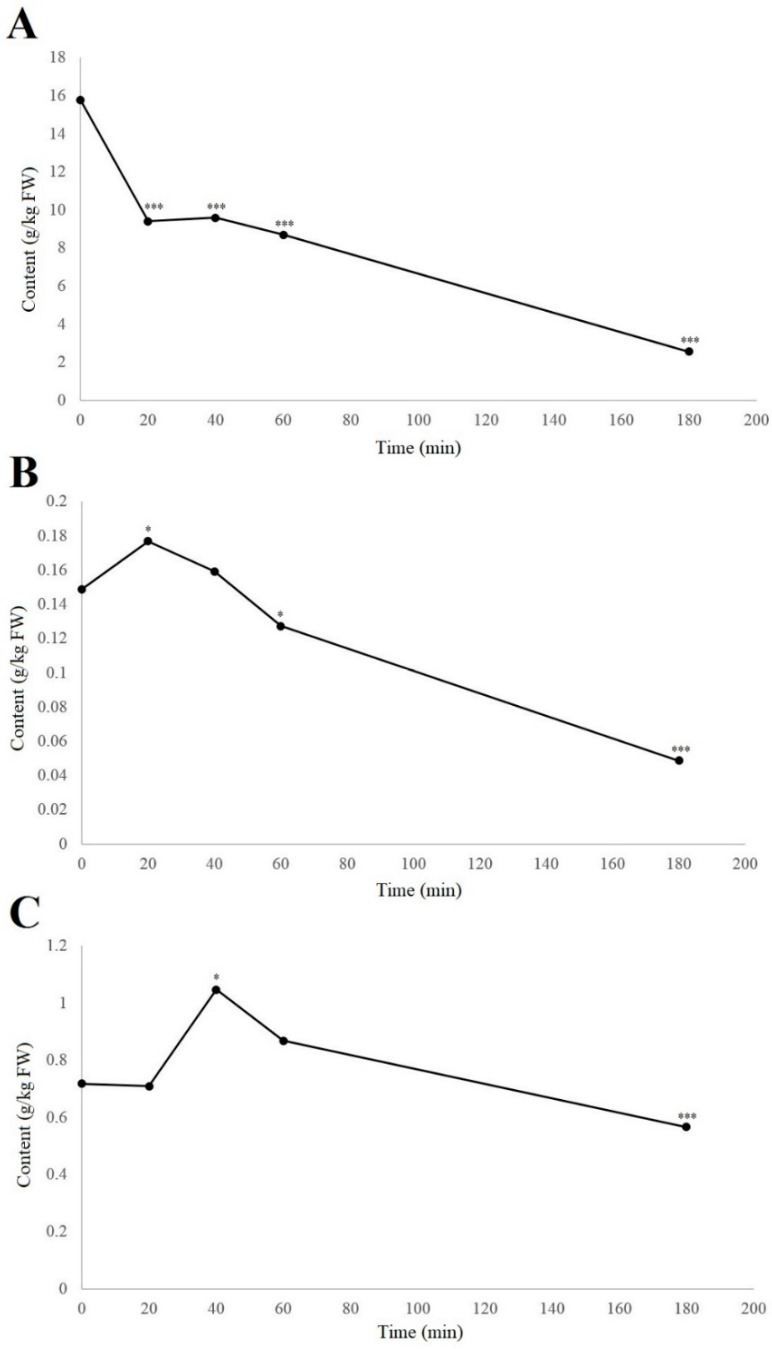
Time-courses of degradation (air, room temperature) of hydrojuglone glucoside (**A**), α-hydrojuglone (**B**), and juglone (**C**) levels in the walnut husk gratings. Data are means ± standard errors (*n* = 4). * *p* < 0.05; *** *p* < 0.001 versus 0 h control (ANOVA, with Dunnett’s test).

When considering the individual phenolic compounds hydrojuglone glucoside, α-hydrojuglone, and juglone, these were examined in more detail because their relationship has been unclear. Furthermore, studies have been contradictory in terms of whether hydrojuglone glucoside and α-hydrojuglone are the precursors for juglone formation [8] or whether there are other precursors from which juglone is formed [23,24]. With the use of UHPLC–MS, their levels over time can be better determined, as even without enzymatic studies, the content of the precursors should decrease following oxidation, while the juglone content should increase, even if only for a short period of time. The time-courses for the degradation in air at room temperature of hydrojuglone glucoside, α-hydrojuglone, and juglone in the walnut husk gratings are shown in Figure 2.

From Figure 2, it can be seen that degradation of the hydrojuglone glucoside, α-hydrojuglone, and juglone in the walnut husk gratings was relatively rapid, with most changes occurring in the first 60 min. Hydrojuglone glucoside degradation was relatively high over the first 20 min, as a decrease from 15.8 to 9.4 g/kg fresh weight (FW), representing a significant 40.5% loss (*p* < 0.001) (Figure 2A). The degradation then slowed to a near minimum of 2.6 g/kg FW by 180 min. Figure 2B,C shows that the rates of degradation of α-hydrojuglone and juglone were different from hydrojuglone glucoside. Here, α-hydrojuglone increased from 0.15 to 0.18 g/kg FW in the first 20 min (+20.0%; *p* < 0.05), and then gradually decreased to the minimum level of 0.02 g/kg FW by 360 min. For juglone, after an initial delay with no significant change (0.7 g/kg FW, 0–20 min), it significantly increased to 1.05 g/kg FW (+47.9%) from 20–40 min, before decreasing to its near minimum of 0.57 g/kg FW by 180 min. As hydrojuglone glucoside content, in 20 min, decreased by 6.4 g/kg (1.9 × 10^−2^ mol), while the content of juglone, in 40 min, increased only by 0.03 g/kg (1.7 × 10^−4^ mol), there was the possibility that other additional degradation pathways of hydrojuglone glucoside were possible, as less juglone is synthesized than the one expected.

**Figure 3:**
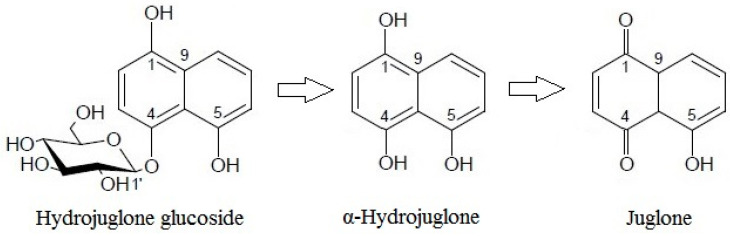
Proposed pathway for juglone synthesis.

These dynamics, whereby hydrojuglone glucoside initially decreased rapidly while α-hydrojuglone increased more slowly, accompanied by a delayed increase in juglone, indicate that Duroux et al. [8] were correct in their prediction of the juglone synthesis pathway. Thus, hydrojuglone glucoside and α-hydrojuglone indeed appear to be the precursors of juglone, in this order (Figure 3). However, enzymatic studies are still needed to confirm this pathway of juglone synthesis. All of the data for the time-courses of degradation of the further individual compounds are given in Supplementary Material Table S1. As can be seen in different phenolic groups, the content of all individual phenolic compounds decreased with time (Supplementary Material Table S1).

### 3.3. Degradation of Different Phenolic Groups over Time

**Figure 4:**
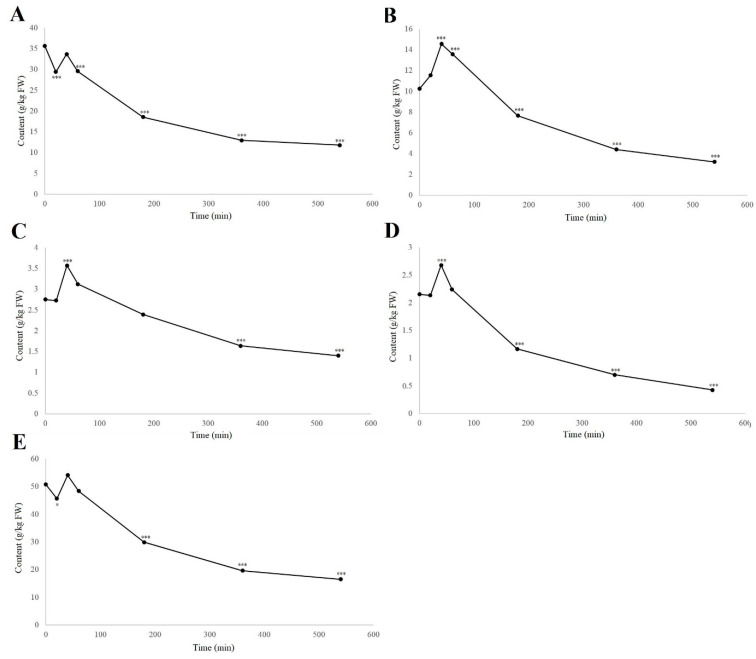
Time-courses of degradation (air, room temperature) of the groups of total naphthoquinones (**A**), flavanols (**B**), flavonols (**C**), and hydroxycinnamic acids (**D**), and for the total analyzed phenolics content (**E**), in the walnut husk gratings. Data are means ± standard errors (*n* = 4). * *p* < 0.05; *** *p* < 0.001 versus 0 h control (ANOVA, with Dunnett’s test).

When considering the groups of phenolic compounds, the time-courses of four phenolic groups were followed, as total naphthoquinones, flavanols, flavonols, and hydroxycinnamic acids. The analysis of the hydroxybenzoic acids is not included here as this ‘group’ contained only one example. The others are shown in Figure 4, along with the dynamics of the total analyzed phenolics content.

As can be seen from Figure 4, these different phenolic groups showed different degradation rates; however, all phenolic groups, and the total analyzed phenolic compounds, reached the highest levels at 40 min of degradation (Table S1). The total naphthoquinones significantly decreased from 35.7 to 29.4 g/kg FW from 0 to 20 min (–17.5%; *p* < 0.001), which was reversed from 20 to 40 min before a gradual decrease to 12.95 g/kg FW by 360 min. This matches up with the changes in hydrojuglone glucoside, α-hydrojuglone and juglone (Figure 2), which suggested that the precursor content decreased in the first 20 min, followed by the formation of the secondary compounds, whereby the total content increased before being further oxidized or lost [19,20]. The total flavanols showed different dynamics to the naphthoquinones, but were very similar to both the flavonols and hydroxycinnamic acids. Here, the total flavanols, flavonols, and hydroxycinnamic acids showed no significant changes from 0–20 min, and then reached their highest levels after 40 min, before gradually decreasing. This effect has also been seen under different thermal treatments and for pathogen attacks, where the content of the phenolic compounds increased [13,16,17,29]. Thermal treatments also promote the release of bound phenols and lead to an increased free phenolic fraction [16], which appears to be similar to when the plant tissue is subjected to damage and oxidation, as in the present study. Thus, the increase in the phenolic content in the first 40 min might be due to their degradation or to an increase in the free phenols, as previously suggested by Crozier et al. [17] and Stewart et al. [18].

As phenolic compounds are considered to be highly beneficial for human health [33,34], this study might have a broader impact. The content of the phenolic compounds in food is believed to decrease when the food is processed or damaged (e.g., cut, bruised) [35]; however, as we see here, instead of decreasing, the phenolic compounds increased due to the breakdown of phenols or an increase in free phenols [17]. Moreover, this increase was not indefinite, but time dependent. Therefore, several studies have reported increased phenolic compounds during thermal treatments [13,16,17], while others have reported decreases [14,15], which indicates that further studies should be conducted to determine whether this is due to the type of food processing or to the timing of the processing method, as proposed. If this is true, ‘correctly’ treated foods could have higher phenolic content than unprocessed foods, as previously suggested by Minatel et al. [35], which could lead to healthier food preparation processes in the future.

## 4. Conclusions

A total of 26 phenolic compounds were identified and quantified, of which, hydrojuglone glucoside, α-hydrojuglone, and juglone were identified in detail using UHPLC–MS. Hydrojuglone glucoside, α-hydrojuglone, and juglone were studied in more detail because their relationships to each other are unclear and studies have been contradictory as to whether hydrojuglone glucoside and α-hydrojuglone are truly the precursors for the formation of juglone or whether there are other precursors from which juglone is formed. We suggest that hydrojuglone glucoside and α-hydrojuglone are indeed the precursors of juglone, but further enzymatic studies should be performed to confirm this definitively. Interestingly, different phenolic groups have shown different degradation processes, but all phenolic groups reached their highest content 40 min after tissue damage and degradation. This increase in phenolic content in the first 40 min might be due to oxidation of inactive forms of phenols that were bound with sugars, an increase in free phenols, or through the plant defense mechanism due to the damaged tissue, similar to stress or pathogen attacks. It has been indicated that the phenolic compounds decrease when foods are processed or damaged, but instead, here, the phenolic compounds increased. This increase was not indefinite, but time dependent. As phenolic compounds are considered to be highly beneficial to human health, this study might have broader impacts, as it indicates the need for further investigations into healthier food preparation processes for immediate consumption. This is the first study on the degradation pathways of juglone using a mass spectrometer, in which we suggest that hydrojuglone glucoside and α-hydrojuglone are indeed the precursors of juglone. However, it is possible that there are other degradation pathways of hydrojuglone glucoside, since less juglone is synthesized than expected. For future studies, isolation of hydrojuglone glucoside is inevitable, to further test whether juglone is the only product compound or whether there are other degradation products formed from hydrojuglone glucoside. As seen with the formation of juglone, the amounts are small and it may be difficult to confirm their structures by NMR.

## Figures and Tables

**Table 1:** Tentative identification by mass spectrometry fragmentation of the 26 phenolic compounds from the husks of *Juglans regia* L., and the standards that were used for their quantification.

Phenolic	Retention Time (min)	(M − H)^−^ (*m*/*z*)	MS^2^ (*m*/*z*)	Quantification Standard
Naphthoquinones				
Dihydroxytetralone hexoside	13.04	339	159, 177	Juglone
Hydrojuglone glucoside	16.26	337	175	Juglone
Hydrojuglone derivative pentoside 1	17.98	435	303, 285	Juglone
Hydrojuglone derivative pentoside 2	18.26	435	303, 285	Juglone
Trihydroxytetralone derivative	19.06	491	271, 331	Juglone
Hydrojuglone rhamnoside	20.64	321	175	Juglone
Trihydroxytetralone galloyl hexoside	20.76	507	331, 271	Juglone
Hydrojuglone derivative pentoside 3	21.27	435	303, 285	Juglone
α-Hydrojuglone	28.21	175	131, 147, 157	Juglone
Juglone (5-hydroxy-1,4-naphthoquinone)	29.99	173	155, 145, 129, 111	Juglone
Juglanin B	31.37	327	312, 253	Juglone
Flavanols				
Procyanidin dimer 1	10.38	577	425, 407, 289	Procyanidin B1
Procyanidin dimer 2	11.47	577	425, 407, 289	Procyanidin B1
(+) Catechin	12.22	289	245, 205, 179	(+) Catechin
(−) Epicatechin	14.53	289	245, 205, 179	(−) Epicatechin
Procyanidin dimer 3	15.53	577	425, 407, 289	Procyanidin B1
Procyanidin dimer 4	16.89	577	425, 407, 289	Procyanidin B1
(epi) Catechin derivative	20.17	441	289	(+) Catechin
Flavonols				
Quercetin-3-galactoside	20.53	463	301	Quercetin-3-galactoside
Quercetin-3-xyloside	21.56	433	301	Quercetin-3-xyloside
Quercetin-3-arabinopyranoside	21.73	433	301	Quercetin-3-arabinopyranoside
Quercetin-3-arabinofuranoside	22.21	433	301	Quercetin-3-arabinofuranoside
Quercetin-3-rhamnoside	22.43	447	301	Quercetin-3-rhamnoside
Hydroxycinnamic acids				
Neochlorogenic acid (3-caffeoylquinic acid)	9.36	353	191, 179, 135	Neochlorogenic acid
3-*p*-Coumaroylquinic acid	12.01	337	163, 191, 173	Neochlorogenic acid
Remaining phenolic compounds				
Gallic acid derivative	21.99	489	271, 313	Gallic acid

[M − H]^−^, pseudo-molecular ion identified in negative ion mode.

## Data Availability

Part of the data presented in this study are available in the Supplementary Material. The remaining data presented in this study are available upon reasonable request from the corresponding author. The remaining data are not publicly available due to privacy.

## Supplementary Materials

The following are available online at https://www.mdpi.com/article/10.3390/biology11020342/s1. Table S1: Time-course data for the 26 phenolic compounds and the total phenolic group content from the husks of *J. regia*.
