# Supplementary material for: Time-Dependent Degradation of Naphthoquinones and Phenolic Compounds in Walnut Husks
Source: Biology (Basel). 2022 Feb 21;11(2):342. doi: 10.3390/biology11020342 (PMC8869098; doi:10.3390/biology11020342)
Supplement: Supplementary file 1 [file biology-11-00342-s001.zip › biology-1604140-supplementary.pdf]

**Table S1.** Time-course data for the 26 phenolic compounds and the total phenolic groups contents from the husks of *J. regia*.

| Compound | Contents (g/kg FW) according to oxidation time (min) | | | | | | |
|---|---|---|---|---|---|---|---|
| | 0 | 20 | 40 | 60 | 180 | 360 | 540 |
| **Naphthoquinones** | | | | | | | |
| α-Hydrojuglone | 0.15 ± 0.01 cd | 0.18 ± 0.01 e | 0.16 ± 0.00 de | 0.13 ± 0.00 c | 0.05 ± 0.00 b | 0.02 ± 0.00 a | 0.02 ± 0.00 a |
| Juglone (5-hydroxy-1,4-naphthoquinone) | 0.72 ± 0.10 ac | 0.71 ± 0.08 ac | 1.05 ± 0.10 c | 0.87 ± 0.06 bc | 0.57 ± 0.06 ab | 0.46 ± 0.08 a | 0.43 ± 0.02 a |
| Hydrojuglone glucoside | 15.77 ± 0.45 d | 9.40 ± 0.29 c | 9.58 ± 0.07 c | 8.68 ± 0.03 c | 2.55 ± 0.03 b | 1.45 ± 0.09 a | 1.36 ± 0.06 a |
| Hydrojuglone rhamnoside | 3.09 ± 0.08 bc | 2.73 ± 0.11 b | 3.41 ± 0.14 c | 3.14 ± 0.07 bc | 2.62 ± 0.15 b | 1.69 ± 0.07 a | 1.57 ± 0.13 a |
| Hydrojuglone derivative pentoside 1 | 1.21 ± 0.11 b | 1.35 ± 0.12 bc | 1.84 ± 0.19 c | 1.79 ± 0.03 c | 1.11 ± 0.16 b | 0.48 ± 0.03 a | 0.40 ± 0.04 a |
| Hydrojuglone derivative pentoside 2 | 1.12 ± 0.07 ac | 1.36 ± 0.12 cd | 1.76 ± 0.06 e | 1.50 ± 0.04 de | 1.21 ± 0.07 bcd | 0.88 ± 0.08 ab | 0.79 ± 0.07 a |
| Hydrojuglone derivative pentoside 3 | 1.69 ± 0.08 bc | 1.80 ± 0.07 bc | 2.30 ± 0.12 d | 2.05 ± 0.08 cd | 1.58 ± 0.12 b | 1.03 ± 0.07 a | 0.86 ± 0.10 a |
| Dihydroxytetralone hexoside | 2.04 ± 0.11 d | 1.13 ± 0.04 c | 1.26 ± 0.08 c | 1.14 ± 0.01 c | 0.65 ± 0.08 b | 0.24 ± 0.05 a | 0.11 ± 0.01 a |
| Trihydroxytetralone derivative | 6.26 ± 0.11 cd | 7.12 ± 0.22 e | 8.75 ± 0.21 f | 6.89 ± 0.15 de | 5.47 ± 0.16 bc | 5.00 ± 0.22 ab | 4.42 ± 0.17 a |
| Trihydroxytetralone galloyl hexoside | 1.37 ± 0.04 b | 1.25 ± 0.07 b | 1.54 ± 0.11 b | 1.56 ± 0.09 b | 1.32 ± 0.13 b | 0.60 ± 0.04 a | 0.58 ± 0.04 a |
| Juglanin B | 0.55 ± 0.09 a | 0.88 ± 0.08 bc | 0.96 ± 0.12 c | 0.91 ± 0.02 c | 0.78 ± 0.06 bc | 0.52 ± 0.04 a | 0.54 ± 0.08 a |
| **Flavanols** | | | | | | | |
| Procyanidin dimer 1 | 2.03 ± 0.09 c | 1.89 ± 0.10 c | 2.63 ± 0.17 d | 2.31 ± 0.03 cd | 0.96 ± 0.08 b | 0.49 ± 0.09 a | 0.17 ± 0.03 a |
| Procyanidin dimer 2 | 2.26 ± 0.07 c | 2.49 ± 0.08 cd | 3.29 ± 0.14 e | 2.79 ± 0.07 d | 0.86 ± 0.12 b | 0.34 ± 0.05 a | 0.10 ± 0.03 a |
| Procyanidin dimer 3 | 1.13 ± 0.08 c | 1.11 ± 0.07 c | 1.38 ± 0.09 c | 1.34 ± 0.02 c | 0.63 ± 0.17 b | 0.13 ± 0.03 a | 0.03 ± 0.01 a |
| Procyanidin dimer 4 | 1.40 ± 0.13 d | 1.05 ± 0.14 cd | 1.28 ± 0.12 cd | 1.31 ± 0.04 cd | 0.90 ± 0.15 bc | 0.41 ± 0.06 ab | 0.21 ± 0.05 a |
| (+) Catechin | 1.41 ± 0.05 c | 1.54 ± 0.07 cd | 1.84 ± 0.08 d | 1.72 ± 0.14 cd | 0.83 ± 0.10 b | 0.33 ± 0.06 a | 0.14 ± 0.03 a |
| (-) Epicatechin | 1.45 ± 0.09 a | 3.01 ± 0.06 c | 3.59 ± 0.10 d | 3.46 ± 0.06 d | 3.09 ± 0.10 c | 2.52 ± 0.10 b | 2.40 ± 0.01 b |
| (epi) Catechin derivative | 0.59 ± 0.03 c | 0.47 ± 0.04 bc | 0.55 ± 0.04 bc | 0.65 ± 0.03 c | 0.39 ± 0.07 b | 0.19 ± 0.04 a | 0.15 ± 0.02 a |
| **Flavonols** | | | | | | | |
| Quercetin-3-galactoside | 0.46 ± 0.03 c | 0.39 ± 0.02 bc | 0.48 ± 0.02 c | 0.46 ± 0.00 c | 0.33 ± 0.02 b | 0.20 ± 0.02 a | 0.16 ± 0.02 a |
| Quercetin-3-xyloside | 0.46 ± 0.05 bc | 0.53 ± 0.02 c | 0.80 ± 0.03 d | 0.71 ± 0.01 d | 0.52 ± 0.04 c | 0.36 ± 0.02 ab | 0.31 ± 0.03 a |
| Quercetin-3-arabinopyranoside | 1.41 ± 0.06 c | 1.30 ± 0.05 c | 1.68 ± 0.07 d | 1.42 ± 0.03 c | 0.96 ± 0.07 b | 0.59 ± 0.03 a | 0.50 ± 0.02 a |
| Quercetin-3-arabinofuranoside | 0.27 ± 0.02 ac | 0.27 ± 0.01 ac | 0.33 ± 0.01 c | 0.32 ± 0.01 bc | 0.30 ± 0.01 bc | 0.26 ± 0.02 ab | 0.22 ± 0.01 a |
| Quercetin-3-rhamnoside | 0.16 ± 0.02 a | 0.24 ± 0.02 ab | 0.28 ± 0.02 b | 0.22 ± 0.01 ab | 0.28 ± 0.01 b | 0.23 ± 0.02 ab | 0.21 ± 0.02 ab |

| | | | | | | | |
|---|---|---|---|---|---|---|---|
| **Hydroxycinnamic acids** | | | | | | | |
| Neochlorogenic acid (3-caffeoylquinic acid) | 0.49 ± 0.01 ef | 0.44 ± 0.02 de | 0.52 ± 0.01 f | 0.42 ± 0.01 d | 0.26 ± 0.01 c | 0.17 ± 0.01 b | 0.10 ± 0.01 a |
| 3-*p*-Coumaroylquinic acid | 1.66 ± 0.03 d | 1.69 ± 0.03 d | 2.16 ± 0.04 e | 1.81 ± 0.03 d | 0.91 ± 0.06 c | 0.53 ± 0.03 b | 0.33 ± 0.01 a |
| **Hydroxybenzoic acids** | | | | | | | |
| Gallic acid derivative | 0.34 ± 0.03 ab | 0.33 ± 0.03 ab | 0.47 ± 0.04 bc | 0.49 ± 0.01 c | 0.41 ± 0.05 bc | 0.23 ± 0.01 a | 0.22 ± 0.01 a |
| Total napthoquinones | 35.66 ± 0.49 d | 29.42 ± 0.55 c | 33.69 ± 0.66 d | 29.60 ± 0.38 c | 18.57 ± 0.64 b | 12.95 ± 0.61 a | 11.80 ± 0.46 a |
| Total flavanols | 10.26 ± 0.51 c | 11.56 ± 0.47 cd | 14.56 ± 0.69 e | 13.58 ± 0.35 de | 7.66 ± 0.73 b | 4.41 ± 0.35 a | 3.21 ± 0.16 a |
| Total flavonols | 2.75 ± 0.17 bc | 2.73 ± 0.11 bc | 3.57 ± 0.12 d | 3.12 ± 0.03 cd | 2.39 ± 0.13 b | 1.64 ± 0.10 a | 1.40 ± 0.10 a |
| Total hydroxycinnamic acids | 2.15 ± 0.04 d | 2.13 ± 0.04 d | 2.68 ± 0.05 e | 2.24 ± 0.04 d | 1.17 ± 0.07 c | 0.70 ± 0.05 b | 0.42 ± 0.01 a |
| **Total analyzed phenolic compounds** | **50.74 ± 1.16 cd** | **45.65 ± 0.80 c** | **54.08 ± 1.44 d** | **48.35 ± 0.76 c** | **29.90 ± 1.57 b** | **19.61 ± 1.10 a** | **16.54 ± 0.75 a** |

Data are means ± standard error; Data with different letters within the same row (i.e., sampling time) are significantly different (P <0.05)
